# Patient Involvement in Geriatric Care – Results and Experiences from a Mixed Models Design Study within Project INTEGRATE

**DOI:** 10.5334/ijic.2517

**Published:** 2018-02-28

**Authors:** Joern Kiselev, Kadri Suija, Marje Oona, Eva Mellenthin, Elisabeth Steinhagen-Thiessen

**Affiliations:** 1 Geriatrics Research Group, Department for Aging and Technology, Charité Unversity Medicine, Berlin, DE; 2 Department of Family Medicine, Institute of Family Medicine and Public Health, Faculty of Medicine, University of Tartu, Estonia, EE; 3 Division of Gastroenterology and Nephrology, Charité University Medicine, DE

**Keywords:** patient involvement, geriatrics, integrated care, shared-decision-making, health literacy

## Abstract

**Introduction:**

Patient involvement is a core component of an integrated care approach. While the benefits and prerequisites of patient involvement have been described in general and additionally for some target populations, little is known about the views and experiences of older people regarding this matter.

**Methods:**

A study with a mixed-methods design was conducted to gain a better understanding about patient involvement in geriatric care. A questionnaire on shared decision-making was administered within a group of older adults in Germany. Additionally, 7 focus groups with health professionals and geriatric patients in Germany and Estonia were held to deepen the insight of the questionnaire and discussing experiences and barriers of patient involvement.

**Results:**

Older people without an actual medical problem expressed a significantly higher desire to participate in shared decisions than those requiring actual medical care. No significant differences could be found for the desire to be informed as part of the care process. No correlation between patients’ desire and experiences on shared decision-making could be observed. In the focus groups, patients demanded a comprehensive and understandable information and education process while the health professionals’ view was very task-specific. This conflict led to a loss of trust by the patients.

**Conclusions:**

There is a gap between patients’ and health professionals’ views on patient involvement in older people. The involvement process should therefore be comprehensive and should take into account different levels of health literacy.

## Introduction

Over the last decade, patient involvement has become more important in health care and was subject to scientific and political debate [1]. The World Health Organization (WHO) World Alliance for Patient Safety is actively emphasizing the role that patients and their families could play in the improvement of health care [2].

However, an exact definition of what constitutes patient involvement of remains unclear [3,4]. Additionally, terms like “patient involvement”, “patient engagement”, “patient empowerment” or “patient centeredness” are often used synonymously when looking into the published literature, leading to further confusion. Vahdat et al. (2014) defined patient participation as an involvement of the patient in the process of decision-making or expressing opinions about different options in terms of treatment methods, sharing information, emotional state and signs of accepting health team instructions [5]. Other definitions like the one of the Center for Advancing Health (CfAH) emphasizes patient responsibility [6]. This approach, however, has two major flaws. First, it does not take into account the patients’ perspective. Second, this definition neglects the responsibility of the health professional and his responsibility for the patient from a professional and legal standpoint. This was reflected by results of the Eurobarometer Qualitative Study on Patient Involvement, demonstrating different views from patients and medical doctors regarding the general understanding what patient involvement is, how to approach it and what benefits could be gained for both sides [7].

It is therefore obvious that any definition of patient involvement is dependent on the subjective viewpoint of patients and health professionals as well as several potential subgroups. For Hogg (2007), the key challenge in the process of planning and provision of patient-oriented health care services is to include opinions and preferences of patients, caregivers and the community to improve health care systems and to gain public approval and confidence [8]. Mead and Bower (2000) provided a model of patient-centeredness with five dimensions of doctor and patient behavioral interaction [9]: the biopsychosocial perspective, the ‘patient-as-person’, sharing power and responsibility, the therapeutic alliance, and the ‘doctor-as-person’, with factors like professional context, communication and personal factors influencing this interaction.

As patient involvement is gradually accepted as a key component of the health care processes and advocated as a means to improve patient safety [10], this development has to include older people and geriatric patients. Lyttle and Ryan (2010) identified seven themes of older patients’ participation [11]: the concept of participation, the desire for older people to be involved, autonomy and empowerment, patients’ expectations, benefits of participation, factors influencing participation; and prerequisites to participation. Their findings suggest that patient involvement is possible when patients are informed accordingly, involved based on their individual knowledge, level of desire to participate, and explanations are understandable. However, little is known on the current state of patient involvement for older people. This is especially true for the health system of Germany, where patient involvement is advocated, but initiatives on the policy context are still sparse [12].

As part of ‘Project INTEGRATE’ [13], a European-wide project on integrated care, we therefore conducted a study on patient involvement for older people using a mixed methods design, incorporating quantitative and qualitative components. As shared decision-making is a core concept of integrated care, we used this concept as a starting point of our research [14,15]. Research was done in Germany and Estonia. The aim of this study was to gain a better understanding on which components of patient involvement are important for patients and/or health professionals, how these components can be implemented in integrated care and which barriers have to be addressed in order to successfully involve a patient in his care process. The results of this study should assist and enable managers and policy makers in the European Union as well as in other countries in the planning and implementation of integrated care in different settings and health care systems.

## Methods

### Theoretical framework

In 2015, Dent and Pahor described a theoretical framework of patient involvement with three components labeled “Choice”, “Co-Production” and “Voice” [16]. Choice refers to the access of information to enable an informed choice or a shared decision within a health-related process while the component of Co-Production represents the ability of the patient to participate in his care process. The third component, Voice, covers the ability and access to advance the health system itself within community-driven projects and platforms. While the third component was not part of our research, Choice and Co-Production are important factors in the care provision in order to enable an environment in which the patient is able to participate in his care process. We therefore used these two factors within this framework for our research.

### Data collection

For our study, we used a mixed methods approach consisting of two parts. In the first part, we administered a questionnaire on attitudes and experiences on shared decision-making within a group of older adults in Germany. In a second step, we organized focus groups in Berlin, Germany and Tartu, Estonia, to discuss topics on patient involvement. We chose this approach as we anticipated that the results from the questionnaires alone would not be able to allow for evaluating the reasons for these findings. The second part of our research aimed therefore at elaborating the results obtained from the questionnaires as suggested by Brannen (2005) [17].

For the quantitative part, we used the Autonomy-Preference-Index (API) [18] and the Shared Decision-Making Questionnaire (SDM-Q9) [19] as standardized tools for questioning two groups of older people. The first group consisted of older people without dependencies in their activities of daily living and no need for acute medical care (disability-free (DF) group); the second group consisted of older people with chronic conditions and comorbidities within a geriatric hospital (GH group). The minimum age for participation was 60 years in both groups. Exclusion criteria were severe cognitive impairment, language barriers, and no written consent (for the GH group). Participants of the DF group were recruited through a senior university program and a sports facility for elderly people; participants in the GH group were recruited in a geriatric hospital.

The API is a standardised questionnaire for the evaluation of attitudes towards participation and information in medical decisions [18]. The API differentiates between a desire to participate in a shared-decision-making process and being informed about the medical process. All items are answered on a 5-point-Likert-scale ranging from “strongly agree” to “strongly disagree”. We used a translated and validated version of the API, consisting of 11 items measuring the same dimensions as the original scale [20]. The Shared Decision Making Questionnaire (SDM-Q) consists of two versions for patients and medical doctors where they describe a situation in which they talked to their counterpart about a medical decision to be made and answer 9 items on different aspects in a shared Bindestrich process [19]. Each item is rated on a 6-Point-Likert-Scale ranging from “completely disagree” to “completely agree”. In our study, we used the German version of the SDM-Q9 for patients [21].

Additional questions consisted of sociodemographic data, level of education, occupation and medical data. This data included the item “care insurance utilisation” in the context of the German health system. Care insurance is an insurance concept for people with a high level of regular care need, which is covered by public-financed care insurance in Germany. A special examination by a medical doctor evaluates and assigns a level of need, varying between 0 (no need) and 3 (high need). Additional differentiation is possible, e.g. when the care need is based mostly on cognitive decline.

During the qualitative part of our study, we conducted six focus groups, three with patients and three with health professionals. The focus groups involving patients consisted of three participants each and were held in Germany (2) and Estonia (1). Two more focus groups with health professionals were held in Germany and one in Estonia. Overall, we included three medical doctors, five nurses, four therapists, a clinical psychologist and a social worker. Estonia was chosen as additional case site for two reasons. First, as partners in Project INTEGRATE we had direct access to the infrastructure and contacts the University of Tartu was able to provide. Second, the health system in Estonia shows many similarities to that of Germany [22]. Therefore, we were able to compare interviews from both countries for any differences and similarities in order to validate the generalizability of the results from the qualitative part of our research.

The interview guideline was developed based on a common methodological approach developed as part of Project INTEGRATE [23] for the basic themes to discuss in the groups. Additional questions were developed based on the results of the quantitative part of our study to elaborate the results obtained there. Further refinement was done by reviewing existing publications on patient involvement in geriatric care.

The final interview guide resulted in the following topics:

1. Importance and experiences with shared decision-making.
2. Prerequisites for a shared decision-making process.
3. Prerequisites for a structured patient-education process.
4. Prerequisites for self-management of a chronic disease.
5. The role of different health professions in shared decision-making, patient education and self-management of the chronic condition(s) by the patients.

Focus groups conducted in Germany took place within the hospital sector; the focus groups in Estonia provided insights in the ambulatory family medicine sector. Interviews took between 45 minutes and one hour. Over the course of each interview, we verbally summarized important points and messages to ensure a clear understanding of the interviewees.

Participants in Germany consisted of patients from a geriatric hospital. The hospital provides geriatric care with an integrated care approach. All health professionals were working in a multiprofessional team and received special education to account for the special needs of geriatric patients. The patients in this setting are mainly older people with multiple chronic conditions. The hospital has 192 beds and treats nearly 300 patients per year. Most common main diagnoses are fractures, cardiovascular diseases included stroke, chronic pulmonary diseases and cancer. Participating family doctors in Estonia were recruited through personal contacts from the Institute of Family Medicine and Public Health, University of Tartu; patients were subsequently recruited through the interviewed family doctors. All participants received written and verbal information about the aims of the study and their role within it. Participation of subjects not admitted to the geriatric hospital as well as the health professionals was anonymous and required therefore no written consent; participation of patients in the geriatric hospital signed a formal written consent for participation. Both parts of this study were conducted to the principles of the declaration of Helsinki and the good clinical practice and received approval from both the ethical committee of the Charité Berlin and from the University of Tartu.

## Analysis

Descriptive statistics of the questionnaires for all participants as well as both groups included tests for normal distribution using the Kolmogorov-Smirnov test (KS) and the Shapiro-Wilk-test (SW). For normal distributed data, mean values as well as standard deviations were calculated and medians for non-normal distributions. For comparison between the two groups of older adults, student’s t-test or Chi-square test was performed, depending of the scaling of the data. Correlation between the desire to participate and participation experience of the patients was tested with a linear regression analysis.

Analysing the focus group interviews was done with a qualitative approach. Interviews were conducted in the respective native language of the interviewees, audiotaped and transcribed verbatim; and then translated into English. A first preliminary analysis was done according to the method provided by Halcomb et al. [24] to identify relevant topics, which were encoded to provide standardisation. For this purpose, the researcher who was assigned to conduct all interviews, was taking notes over the course of each interview. After each interview, he then journalized them immediately based on these notes before listening to the audio tape for reviewing and amending the notes. Finally, a preliminary content analysis was performed and a first set of codes were derived from this analysis. Following that, a second researcher transcribed the interviews before both researchers performed a content analysis [25] to derive a final set of codes. Consensus was reached by comparing and discussing results. Over the course of the analysis, the coding list was further adapted if necessary to ensure the inclusion of all relevant topics.

Finally, we integrated both parts of our study and discussed implications following our results in an expert meeting involving different members from Project INTEGRATE within a regular biannual meeting over the course of the project.

## Results

### Quantitative part: Questionnaires

We were able to obtain 141 questionnaires from the DF-group and 142 questionnaires from the GH-group. Of those, 15 questionnaires had to be excluded because of incomplete data. We considered a questionnaire as incomplete if both the API and the SDM-Q9 were not filled out completely. Sociodemographic data of all participants are provided in Table 1. Of the participants, 95 were male and 187 female with no significant differences between both groups. Average age of participants differed significantly between both groups with the GH-group being significantly older. We did not find significant differences for family or housing status.

**Table 1:** Sociodemographic data of participants in the quantitative phase of the study.

Participants		DF		GH		Total		p-value

Number of participants		N = 141		N = 142		283		

		N	N%	N	N%	N	N%	

**Age (years)**		Mean = 72 (SD = 6)		Mean = 77 (SD = 7)		Mean = 74.42 (SD = 7.26)		>0.001
**Gender**	Male	46	33.3%	49	34.0%	95	33.6%	
	Female	92	66.7%	95	66.0%	187	66.1%	n.s.
**Family status**	Single	10	7.2%	16	11.1%	26	9.18%	
	Married	68	49.3%	59	41.0%	127	44.9%	
	Widowed	27	19.6%	34	23.6%	61	21.6%	
	Divorced/Living apart	32	23.2%	32	22.2%	64	22.6%	n.s.
	Non-marital partnerships	0	0.0%	3	2.1%	3	1.1%	
	Missing	1	0.7%	0	0.0%	1	0.4%	
**Housing**	Private home	134	97.1%	137	95.1%	271	95.8%	
	Assisted living	3	2.2%	4	2.8%	7	2.5%	
	Retirement and nursing homes	1	0.7%	1	0.7%	2	0.7%	n.s.
	Missing	0	0.0%	2	1.4%	2	0.7%	
**School Education**	None	0	0.0%	3	2.1%	3	1.1%	
	Primary school	25	18.0%	50	34.7%	75	26.5%	
	Secondary school	25	18.0%	50	25.7%	62	21.9%	
	Specialized secondary education	23	16.5%	13	9.0%	36	12.7%	>0.001
	Vocational secondary education	66	47.5%	39	27.1%	105	37.1%	
	Other	0	0.0%	1	0.7%	1	0.4%	
**Professional Education**	No	1	0.7%	21	14.6%	22	7.8%	
	Professional training	30	22.1%	65	45.1%	95	33.6%	
	Advanced professional training	28	20.6%	17	11.8%	45	15.9%	>0.001
	University of Applied Sciences degree	26	19.1%	20	13.9%	46	16.3%	
	University degree	48	35.3%	20	13.9%	68	24.0%	
	Other	3	2.2%	1	0.7%	4	1.4%	
**Occupational status**	Employee	94	68.1%	106	74.6%	200	70.7%	
	Civil Servant	17	12.3%	11	7.7%	28	9.9%	
	Self-employed/freelancer	11	8.0%	11	7.7%	22	7.8%	n.s.
	Home keeper	7	5.1%	11	7.7%	18	6.4%	
	Other	9	6.5%	3	2.1%	12	4.2%	

Abbr. DF: Disability-free; GH: Geriatric Hospital; N: Number of participants; n.s.: non-significant; SD: standard deviation.

For school and professional education, we asked about the highest level achieved. Occupational status referred to any current occupation participants were holding or the last occupation in case of retirement. Levels of educational status and professional qualification were significantly higher in the DF-group compared to the GH-group. However, occupational status was not significantly different between the groups (Table 1).

The number of diseases and chronic conditions differed not significantly between both groups. However, number of medications as well as care insurance utilisation and levels of disability were significantly higher in the GH-group. Additionally, a significant difference in the subjective perception of health could be observed with the GH-group having a higher percentage of perceiving their health as “Bad” or “Not Well” and the DF-group having a higher percentage of those perceiving their health as “Good” or “Very Good” (Table 2).

**Table 2:** Objective and subjective health status of the participants.

Participants		DF		GH		Total		p-value

Number of participants		N = 141		N = 142		283		

		N	N%	N	N%	N	N%	

**Care Insurance Utilization**	Yes	2	1.5%	39	27.3%	41	14.5%	>0.001
	No	131	97.0%	89	62.2%	220	77.8%	
	Applied for	1	0.7%	12	8.4%	13	4.6%	
**Disability**	Yes	101	74.3%	65	45.1%	166	58.7%	>0.001
	No	32	23.5%	74	51.4%	106	37.5%	

**Number of medications**		M = 2 (SD 2)		M = 9 (SD 5)		M = 11 (SD 7)		>0.001
		MD = 2		MD = 9		MD = 11		

**Number of diseases and chronic conditions**		M = 4.49 (SD 3.34)		M = 5.06 (SD 3.56)		M = 4.78 (SD 3.46)		n.s.
		MD = 4.00		MD = 4.50		MD = 4.00		

**Subjective perception of health**	Bad	4	3.0%	35	24.5%	39	13.8%	>0.001
	Less well	19	14.3%	75	52.4%	94	33.2%	
	Good	92	69.2%	32	22.4%	124	43.1%	
	Very good	18	13.5%	1	0.7%	19	6.7%	

Abbr. DF: Disability-free; GH: Geriatric Hospital; M: mean value; MD: median value; N: Number of participants; n.s.: non-significant; SD: standard deviation.

Results of the API for both groups are presented in Table 3. As can be seen, the desire for participation was only moderate for all participants (40.44, SD 27.41) while the desire to be informed was very high (94.37, SD 10.46). A significant difference though was observable between the two groups (DF vs. GH) with the DF group expressing a significantly higher desire to participate in a shared decision-making process. For the desire to be informed, no significant differences could be detected.

**Table 3:** Participation and information desire in both groups.

Participants	DF	GC	Total	p-value

**Desire for participation (Mean/SD)**	47.02/26.40	32.05/26.47	40.44/27.41	<0.001
**Desire for information (Mean/SD)**	93.75/11.86	95.15/8.40	94.37/10.46	n.s.

Abr.: DF: Disability-free; GH: Geriatric Hospital; n.s.: non-significant; SD: Standard Deviation.

**Figure 1:**
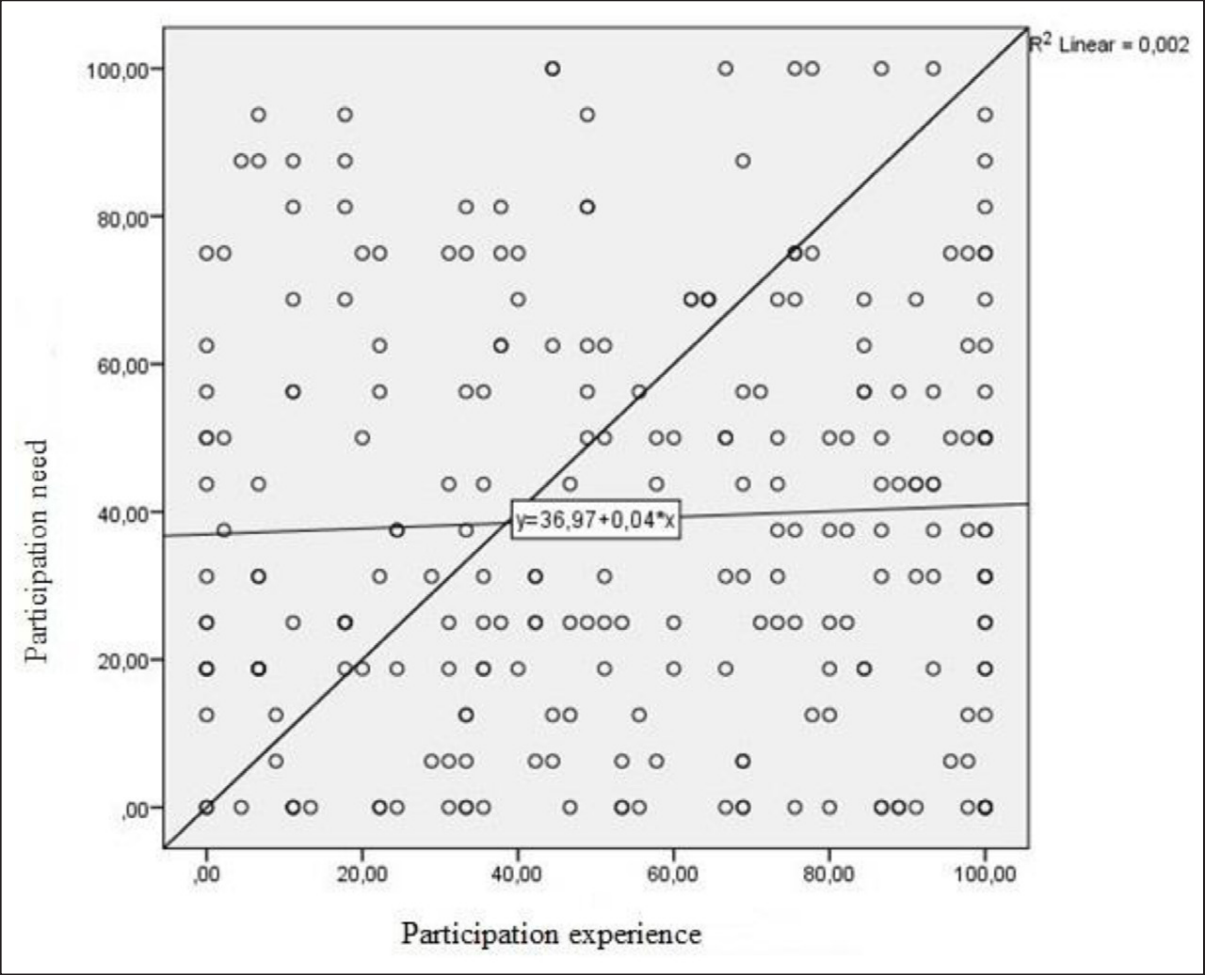
Correlation between participation desire and experience of all participants.

Finally, we conducted a linear correlation analysis between the stated desire for participation of the participants based on the results of the API and the perceived involvement in a shared decision-making process based on the results of the SDM-Q9. The linear correlation between these two factors can be seen in Figure 1, showing no significant correlation between desire for participation and participation experience by the participants.

### Qualitative part: focus groups

#### Patients

The analysis of the interview with the patients revealed four topics as part of the coding process: Prerequisites for involvement in the medical process, getting appropriate and understandable information, patient education and care process organisation.

##### Prerequisites for involvement in medical decisions

While most patients agreed that they wanted to be involved in their medical process, one patient stated he was glad that their doctor just told him what had to be done. *“If I would have known that information before the start of that procedure, I would have thought about starting this at all. […] Therefore, I am glad that the doctor did not tell me this piece of information”* (Patient 3). All patients agreed, however, that in order to be involved, they needed appropriate and understandable information from the medical doctor. For this, one participant phrased very precise demands: *“To be informed on his condition, what his situation is and what steps are next. What can I do, what can be my role, how should be my behaviour.”* (Patient 2). A concern of the patients was that medical doctors would influence conversations on medical decisions in order to manipulate patients to agree to their preferred procedure. *“Doctors are pushing to do what they think is the best. […] So you’re totally dependent on the doctors’ opinion. If he says it’s the only chance, what can I do?”* (Patient 1). This discredited, according to one patient, the whole participation process. *“I would not like to get the impression that the doctor is only informing me in detail because he wants to safeguard himself and not enable me to make my own decisions. And I can’t understand fully why he is giving me the kind of information.”* (Patient 3). On the other side, good experiences with the doctor and good results from treatments were able to build trust. “*If things go the way the doctor says they will, you trust them.” Patient 7*).

#### Getting appropriate and understandable information

The participants had very precise notions on the available information throughout the medical process. Information should be processed in a way that made the information process easy to understand. *“The doctor should explain things in plain language which I can understand and not in his professional language. […] Sometimes, I have to ask for this. […] You have to be able to agree on the procedure the doctor is suggesting.”* (Patient 6). This was especially important for medication regime. *You’re totally dependent on the doctor and his sharing of information.”* (Patient 1). Trust was seen as the central requirement for a satisfactory information process. *“I think you have to be able to rely on the doctors’ opinion. You have to have a certain level of trust.”* (Patient 6). One patient in particular expressed additionally the desire to remain in control of his situation: *“But the question is: Can I control what I get prescribed? Is this what I need? When they change medication, is this still what I really need and on the right time?”* (Patient 3).

##### Examples were given on very good and bad events of patient information and medical interventions

*“The hospital where I had surgery invited all patients they would treat. They introduced the surgeon, the therapists, and the leading nurse. Then, they informed us about the whole procedure, about everything what was ahead of us. And then we had face-to-face conversations with the doctor and the other medical staff. We were there practically the whole day, had a meal there and had the opportunity to take a look at the facility, especially the therapeutic rooms.”* (Patient 3). Such a structured and comprehensive informational approach seemed to be able to build the trust needed for the patient to feel comfortable. *“You develop an enormous level of trust if you get that many information and everything is open to you and when you have the feeling to understand everything.”* (Patient 3).

In contrast, an inappropriate information process left one patient ‘shocked’. *“When I had surgery on my lung, that was awful. First, they were telling me that they were removing only a small part of the lung to see what it is […]. And then, the nurse came to me late in the evening and was telling me that the professor had seen the pictures again, you have to sign this for me, you have cancer. I was so shocked.”* (Patient 2).

#### Structured education process

Two patients reported on structured education processes which they deemed as very important for them and their ability to self-manage their condition. *“And this helps a lot. When I have distress, I know what to do*.” (Patient 5). The structured process was seen as important for both patients. Both stated that without this education class, they were not able to manage their condition as they were now. *“But now, with this this whole education process, they took away the fear that something could happen. This is gone.”* (Patient 5). In comparison, self-education was seen more sceptical. *“When you read it, you are maybe missing something important.”* (Patient 6).

Another very important point in the educational process for managing the chronic conditions was the involvement of family members. *“I would like to have family member to be included in such education classes. My husband has diabetes and once we went home and suddenly he started to behave very strangely. Had very empty eyes. And I had no clue what was happening until I got it. The sugar level! Then I sat him at a table and made something to eat. And then, everything was good again. And for this, you should include family members in education classes.”* (Patient 6).

### Organisation of the care process

The patients saw two very central issues within the organisation process within the hospital which acted as a barrier for the involvement of patients. The first issue was time. As one patient expressed, the whole process of informing and involving the patient takes time which the medical doctor often does not have. *“They have no time to explain things properly and that makes things difficult.”* (Patient 2). The second issue was the perceived level of competence of the hospital staff when the patients perceived deviations from the procedures they were used to self-manage themselves. *“I have diabetes and can manage it well, I had several education classes. And now, I always had white bread on my plate. […] It took me years to get used to dark bread, but it was nowhere to get.”* (Patient 6). This was, according to one patient, a frequent problem. *“I’ve been through a number of hospitals and nowhere was I able to get what I wanted.”* (Patient 6).

### Health professionals

Four topics arose from the conducted interviews: Shared decision-making, patient education, self-management and health professional education.

### Shared decision-making

All health professionals agreed on the importance of a shared decision-making process in general. One of the therapists pointed out: *“I think it is essential. We have to work for a mutual goal and it doesn’t work if the therapist sets himself some functional goal and is obtruding this goal on the patient and the patient is pursuing other goals which are important for his daily life. In the worst case, you work separately, everyone on his goal and in the end, you achieve nothing.”* (Therapist 2).

The shared decision-making process was described by two medical doctors basically the same way. *“First I describe the situation to the patient so that he has the same level of information as I have. After that; I openly ask him about his preferences, what he thinks would be his choice. I compare that with my personal preferences and then we talk about it.”* (Doctor 2). However, neither the doctors nor the therapists described a specific approach to this process. *“No, no specific strategy. Patients’ can be so different, their personality and situation can be different. […] You have to have a new strategy every time.”* (Doctor 1). One medical doctor expressed it as ‘recognizing’ the readiness of the patient to be involved: *“At one point you’ll recognise that they’re ready, which is when you offer them that chance. You have to really think about how you suggest they proceed.”* (Doctor 3).

While the interviewed nurses generally agreed on the importance of involving the patient in decisions, some aspects of their work made a shared decision-making process difficult, as for some actions there was no real choice to make. *“Some principles are there, of course. The patient has to take his medication; wounds have to be cared for. But you have to find a way to get it done… […] You are speaking with the patient, telling him what we should do and if not it becomes worse.”* (Nurse 2).

Unwillingness to be involved in a shared decision-making process was perceived by the medical doctors as a matter of interest and attributed to certain diseases. *“Patients with any lack of interest in any change. COPD patients for example. They want to go home as fast as possible and continue smoking. Talks with these patients can be very frustrating.”* (Doctor 1). Another perceived factor contributing to the participation process was age. *“The younger the person is, the more they’re interested in participating in making decisions that concern their state of health. Older clients tend to want specialists to make decisions on their behalf.”* (Doctor 3). In contrast, the interviewed therapists saw a lack of information as the most important barrier for involving patients. *“But most often this fails because the patients have not enough information.”* (Therapist 1). Age was seen as a factor by the therapists too, but the emphasis was more on the patients’ expectations. *“…patients are often times not used to participate, especially the older ones”*. (Therapist 1).

Another barrier on the process of involving the patient in a shared-decision making process was the formal framework in Germany. As certain formal requirements had to be met for a proper imbursement, the choices of the patient were limited. *“We have to do certain therapies in order to bill our treatment as complex. So we can’t ask the patient what he wants.”* (Therapist 1).

### Patient education

For the medical doctors in the geriatric hospital, patient education was a very important part of his relationship to the patient that was mostly embedded in the daily routine. Topics of patient education were almost exclusively to provide task-specific skills for self-managing aspects of their condition. One doctor talked about educating patients with a chronic obstructive pulmonary disease (COPD) patients in the use of inhalers. *“During the visits I ask the patient if he can demonstrate the handling with the device.”* (Doctor 1). However, some of the education tasks were delegated to the nurses. *“I either demonstrate the use or give an instruction to one of the nurses.”* (Doctor 1). The decision on whether educating the patient personally or delegating this to a nurse was mostly the expected time to practice the use. *“For most patients, instruction in the use of the inhaler takes time – about half an hour – which we as medical doctors most often can’t afford, so we delegate this work to the nurses.”* (Doctor 1). For the health professionals in the ambulatory sector, patient education was mostly done by verbal information and by providing written material. *“I give patients materials to take with them so they can look at them at home. But we go through them together as well, so they know what to focus on.”* (Doctor 3). Another way for providing information was to give them information on available websites. *“I’ve directed them to websites where they can get information […]. Then they can get further information at home.”* (Doctor 4). None of the medical doctors was involved in a structured patient education program.

For the therapists, patient education was limited on teaching self-dependent exercises. *“First you have to teach them certain basic abilities and the patient should be at a point where he is able to convert the exercises in his daily routine.”* (Therapist 2). Providing more specific information about the chronic condition of a patient was seen as the responsibility of the doctor. *“I think this is more a doctors’ task.”* (Therapist 1).

### Self-management

In principle, the same approach was chosen for controlling sufficient self-management skills, although it was stated that it was important to get feedback from both the patient and the educating nurse. *“During the next visits by letting the patients do it again or you’re asking the nurse who did the education. You have to ask both sides to have the whole picture.”* (Doctor 2). The same was true for the therapists, who saw ‘self-management’ as a way to incorporate exercises in their daily life and taking responsibility for their body. *“It should be like that the patient knows he’s getting a framework for his own work, so the therapist is showing him his way, his opportunities, but the progress is not expectable in the short sessions of therapy; you have to put additional work into your body in order to be able to expect results. It’s up to you what you make out of it. And I’m not responsible for it when you don’t do it.”* (Therapist 2). Interestingly, while providing knowledge to the patient in order to sufficiently self-manage his condition was not a topic for patient education, it was acknowledged that this was a very important point. *“He has to be educated not only in doing it but also about the reasons why it is so important.”* (Doctor 1).

### Health-professional education

As the importance of patient involvement was generally acknowledged, part of the focus group discussions centred on opportunities on the necessary communication skills and how the skills of the patient can be identified. Both medical doctors from the geriatric hospital complained that patient education and self-management concepts were not part of the University education. *“Unfortunately this is not taught in University.”* (Doctor 2). On the other hand, special courses seemed to be available both in Germany and in Estonia. *“I think that every new in-service training course where you boost your professional knowledge and improve how you work with clients.”* (Doctor 1). In contrast, involving the patient in the decision-making process and teaching them exercises seemed more natural for the therapists. *“It’s the only way I know. I give everyone something to do as homework.”* (Therapist 2).

## Discussion

In our study, we used a mixed-method design combining quantitative and qualitative research approaches to analyse and evaluate the level and extend of patient involvement in the population of older people, but also perceived barriers from the patients’ and the health professionals’ perspective.

Regarding the conducted questionnaire, we were able to draw two conclusions. First, in our cohort of older people living in Germany, those with a current need of intervention had a significantly lower desire to participate in a medical decision process. In comparison, higher levels of the desire to participate in shared decision-making processes in patients with a variety of diagnoses could be seen in a pooled data analysis from six trials [26]. While the cohorts of these trials were all younger than our cohorts, we are not able to interpret reasons for the lower participation desire in our participants. One possible explanation is that the desire of patients to be informed and to understand the underlying process of their condition in order to be willing or able to participate in the decisional process (and all subsequent steps) is getting more difficult with the rising complexity of their condition. This might discourage older people but also discourage health professionals whose responsibility it is to initiate the shared decision-making process. This was reflected by our interviews where the physicians demonstrated the subjective estimation that age would be a factor for the willingness to participate in a decision-making process. Second, we could not detect any correlation between the participants desire to be involved in a shared decision-making process and their experience with medical doctors. This finding was both true for subjects who wanted or not wanted to be involved. The question if this results stem from a lack of awareness or willingness to involve older patients in medical decisions cannot be answered based on the results of the questionnaires. However, none of the health professionals in our interviews described a structured approach for shared decision-making and instead emphasised an individual approach based on empathy and experience. We therefore assume that in our group of health professionals, this lack of a structured approach could lead to false conclusions regarding the patients’ desire to be involved. Structured instruments for the measurement of patient involvement are focussing on the observable extend of patient involvement [27]. While such measurements are important in scientific research, clinical routine demands a practical instrument to recognize the level of willingness to participate in a specific patient. Despite the willingness of health professionals and patients alike to use such instruments [28], none have been developed to our knowledge. Therefore, health professionals are limited to their clinical experience and empathy which can, as could be demonstrated in our work, lead to false conclusions.

Based on the conducted focus groups, several common factors could be identified. One of the central themes in the patient focus groups was that patient involvement would require an appropriate information process. For this, information and education processes needs to be structured in a way where patients are able to understand their own condition, the acute medical interventional process and the required long-term measures. These factors were described by Nutbeam (2000) as part of the term ‘health literacy’ [29]. According to the WHO, health literacy consists of four domains: 1) accessing or obtaining information; 2) understanding relevant information; 3) appraising, judging or evaluating information; and 4) applying or using information relevant to health [30]. In contrast, the interviewed patients decried too much professional language in conversations or written information, especially in their relationship with medical doctors. Additionally, they felt dependant on the willingness of the medical doctor to provide sufficient information. Several patients themselves concluded that a gap of knowledge was unavoidable and therefore they would need the trust that they are getting all relevant information by the doctor. Therefore, getting the impression that the doctor withholds some information would absolutely impair this trust. The last point is especially important as the relationship between the patient and the medical doctor has to be based on trust, as was stated several times in the conversations with patients. Obviously, an insufficient information process and the subjective impression that the medical doctor could withhold any information deemed important to the patient can disrupt this trust. As a consequence, the whole information process and the aim of involving the patient in the care cycle are discredited as “safeguarding”. Interestingly, this conflict was not seen by the health professionals, which could be part of the problem.

All in all, patients did not made; over the course of the discussion, the distinction between information, education and self-management. Instead, these three factors seemed to be perceived as a continuum in which one factor was influencing the others. Health literacy was seen as a central factor imperative to the ability to initiate the other three factors in that continuum. This was demonstrated by two reports on structured patient education programs in which all three aspects were taught as parallel and interrelated to each other. Those patients that did not receive any formal and structured program perceived such programs as opportunities for an improvement of their own self-management skills.

Leenerts et al. (2002) provided a theoretical concept of self-care that consisted of five dimensions for health promotion in independent older adults [31]. The dimensions of this model were internal and external environment, self-care ability, education, self-care activity and outcomes; with the first four dimensions influencing the fifth dimension, outcomes. A successful self-care process requires competencies in all of the first four dimensions; while providing and developing these competencies is part of the responsibility of the different health professions working with the patient. The relationship between patient and health professional was described as “partnership”. According to Leenerts, an approach of partnership ensures a common basis and level of trust between both sides and a higher probability that specific problems and needs of the patient are met.

In contrast to the patients’ perspective, the interviewed health professionals demonstrated a very task-specific attitude to this. They tended to see the information- and/or education process as well as the self-management ability as related to a specific problem or action; in contrast to the requirements phrased by the patients who demanded a complete picture of the process. Additionally, factors like older age and lower levels of health were subjectively associated with a lower willingness to participate in a medical process of a specific patient without describing any identification process of the actual desire of a given patient to participate. As could be seen in our questionnaire study, this obviously leads to an inadequate identification process. On the organisational side, time restrictions were named as the most important barrier for patient involvement.

While our approach aimed at a comprehensive analysis of the extent of patient involvement as well as existing barriers in Geriatrics, some limitations are restricting the generalisability of our results. First, while we took every effort to distribute our questionnaires, we were only able to include 283 complete questionnaires. Because of the recruiting strategy especially for older people without disabilities, we were not able to influence the response rate. This might have resulted in the relatively low rate of participants and to a potential bias in the sociodemographic profile of this group. More, we defined a minimum age of 60 years as an inclusion criterion, which is rather young for geriatric patients. However, with a mean age of over 74 years we think that our sample is rather representative. Additionally, as with all qualitative research, the results are mainly a reflection of subjective viewpoints of a selected group, which further limits the transferability of our results. We aimed at expand our results by providing additional interviews from a different care setting and within a different health system. As we found relatively homogeneous results in our interviews in both sites, we think this underpins the generalisability of evidence our findings. Furthermore, we were not able to include all relevant health professionals in our focus groups at both sites, as in Germany we were not able to recruit social workers and neuropsychologists. These two professions would potentially been able to provide further information and insight. Unfortunately, no members of these professions were able to participate within our given timeframe due to workload issues. Therefore, the results of this study have to be interpreted cautiously within the given context. Finally, we conducted our research in two European countries, Germany and Estonia. So, our results cannot be transferred to other countries with different health systems and potential attitudes towards patient involvement without caution.

However, by incorporating the results from the first part of the study and presenting them to an independent group of patients and health professionals alike we feel confident that we were able to identify some important factors, which can act as either a contributor or barrier against patient involvement in older people. For several findings on the patients’ side like their proposed prerequisites for patient involvement or the development of mistrust based on an inappropriate communication strategy we found congenial concepts on the side of the health professionals. While the impression of ‘safeguarding’ may be exaggerated, it can be comprehended from the patients view. This is an important finding, as trust is one of the most important, if not the most important element in the relationship between patient and health professional. Concepts of involving patients in the medical decision or intervention process have to take into account to retain the level of trust necessary for a partnership approach as proposed by Leenerts et al. (2002) [31]. For this, patients should not only be able to perform certain actions in their self-management process but also understand the implications every step in the medical and care process, including their own role, has on the short- and long-term development of their condition. This makes a comprehensive and structured information and education process necessary which still takes into account the individual needs of each patient. This is especially true for patients with complex conditions. Finally, the self-management ability and the level of success of the self-management process should be supervised. In this, the role of health professionals has to be strengthened; but especially nurses and therapists could play a more pivotal role in this supervision effort.

In our view, the results of this study can help to understand important components and prerequisites on how to involve patients in the planning and conduction of their care process. The enablement of patients to participate in their care process is an important feature of patient-centred care [32].

Without a proper process to involve patients and enable them to make informed decisions and handle their own condition(s), the necessary levels of trust and collaboration are difficult to achieve. As the involvement of patients in the care process is a core component of integrated care [14,33], this has to be considered in integrated care too. Managers and policy makers alike should consider this in their work on implementing components of integrated care into their respective fields of work. Additionally, future studies should emphasise these results and include them in their research to further deepen the insight into the potential of PI in older people and geriatric patients.

## Conclusion

In our study, there was a clear and perceivable gap between the phrased prerequisites for patient involvement by the patients and by the health professionals. While patients demanded a suitable and structured information and education process in order to feel able to participate, health professionals perceived a lacking willingness of a patient to be involved with as a lack of interest and described any education process of a patient very task specific. Additionally, the self-perceived dependency on the medical doctor regarding the provided information was seen as a potential source of a loss of trust in the communication between doctors and patients. Health professionals should be aware of this danger in their approach to patients and provide comprehensive and suitable information over the whole course of any intervention. Health professionals of all sorts have to be able to identify the level of health literacy as well as the desire for participation of their patients in any care process. For this, suitable concepts seem to be lacking and should be developed.
